# GPR88 in A_2A_R Neurons Enhances Anxiety-Like Behaviors

**DOI:** 10.1523/ENEURO.0202-16.2016

**Published:** 2016-08-17

**Authors:** Aura Carole Meirsman, Anne Robé, Alban de Kerchove d’Exaerde, Brigitte Lina Kieffer

**Affiliations:** 1 Département de Médecine Translationnelle et Neurogénétique, Institut de Génétique et de Biologie Moléculaire et Cellulaire, Institut National de la Santé et de la Recherche Médicale U-964, CNRS UMR-7104, Université de Strasbourg, 67400 Illkirch-Graffenstaden, France; 2 Laboratory of Neurophysiology, ULB Neuroscience Institute, Université Libre de Bruxelles, 1070 Brussels, Belgium; 3 Department of Psychiatry, Faculty of Medicine, Douglas Research Center, McGill University, Montréal, Québec H4H 1R3, Canada

**Keywords:** amygdale, anxiety-like behavior, D2R-medium spiny neurons, G-protein-coupled receptors, striatum

## Abstract

GPR88 is an orphan G-protein-coupled receptor highly expressed in striatal dopamine D_1_ (receptor) R- and D2R-expressing medium spiny neurons. This receptor is involved in activity and motor responses, and we previously showed that this receptor also regulates anxiety-like behaviors. To determine whether GPR88 in D2R-expressing neurons contributes to this emotional phenotype, we generated conditional *Gpr88* knock-out mice using adenosine A_2A_R (A_2A_R)-Cre-driven recombination, and compared anxiety-related responses in both total and *A_2A_R*-*Gpr88* KO mice. *A_2A_R*-*Gpr88* KO mice showed a selective reduction of *Gpr88* mRNA in D2R-expressing, but not D_1_R-expressing, neurons. These mutant mice showed increased locomotor activity and decreased anxiety-like behaviors in light/dark and elevated plus maze tests. These phenotypes were superimposable on those observed in total *Gpr88* KO mice, demonstrating that the previously reported anxiogenic activity of GPR88 operates at the level of A_2A_R-expressing neurons. Further, *A_2A_R*-*Gpr88* KO mice showed no change in novelty preference and novelty-suppressed feeding, while these responses were increased and decreased, respectively, in the total *Gpr88* KO mice. Also, *A_2A_R*-*Gpr88* KO mice showed intact fear conditioning, while the fear responses were decreased in total *Gpr88* KO. We therefore also show for the first time that GPR88 activity regulates approach behaviors and conditional fear; however, these behaviors do not seem mediated by receptors in A_2A_R neurons. We conclude that *Gpr88* expressed in A_2A_R neurons enhances ethological anxiety-like behaviors without affecting conflict anxiety and fear responses.

## Significance Statement

GPR88, a striatal enriched orphan G-protein-coupled receptor has been implicated in the regulation of anxiety-like behaviors. In the striatum, GPR88 is most abundant in both medium spiny neurons expressing dopamine D_1_ and D_2_ receptors (Rs). To evaluate the contribution of GPR88 in D_2_R-neurons, we compared anxiety-like and fear-related behavioral responses of newly generated conditional *A2AR*-*Gpr88* mice with those of total *Gpr88* knock-out animals. Our data show that GPR88 expressed in A2AR neurons increases ethological anxiety-like behaviors without affecting conflict anxiety and fear responses. These results represent a first step toward understanding the circuit mechanisms underlying GPR88 function in the brain. Future studies will evaluate the role of GPR88 in D_1_R neurons.

## Introduction

G-protein-coupled receptors (GPCRs) are the target for ∼40% of marketed drugs, and are major players in biomedicine (Rask-Andersen et al., 2011). Orphan GPCRs, whose ligands remain unknown and functions have been little studied, offer great promise (Levoye et al., 2006; Rask-Andersen et al., 2011; Ghanemi, 2015). The orphan GPCR GPR88 has been implicated in a number of behaviors related to psychiatric disorders. Mice lacking *Gpr88* present a complex behavioral phenotype that includes motor coordination deficits, reduced prepulse inhibition, stereotypies, and altered cue-based learning (Logue et al., 2009; Massart et al., 2009; Quintana et al., 2012; Ingallinesi et al., 2015). These behaviors can all be related to the strong enrichment of GPR88 in the striatum (Swerdlow et al., 2001; Lewis and Kim, 2009; Liljeholm and O'Doherty, 2012). In humans, the *Gpr88* gene was associated with bipolar disorders and schizophrenia (Del Zompo et al., 2014). Recently, we found that *Gpr88* deletion in mice also decreases anxiety-like behavior (Meirsman et al., 2016), implicating this receptor in emotional processing and in the evaluation of environmental stimuli value. Concordant with this finding, *Gpr88* expression was shown regulated by antidepressant and mood-stabilizer treatments in both rodent models and humans (Ogden et al., 2004; Brandish et al., 2005; Böhm et al., 2006; Conti et al., 2007).

Several lines of evidence suggest that GPR88 alters behavior by modulating striatal transmission. In the striatum (dorsal and ventral), GPR88 is most abundant in medium spiny neurons (MSNs) expressing dopamine D_1_ receptors (Rs; D_1_R-MSNs coexpressing substance P) and dopamine D_2_R MSNs coexpressing adenosine A_2A_R (A_2A_R) and regulates the excitability of these neurons, possibly by acting on glutamatergic, GABAergic, and dopaminergic receptors activity (Logue et al., 2009; Quintana et al., 2012). Conversely, glutamatergic and dopaminergic depletion differentially alters *Gpr88* expression in these distinct MSN subpopulations (Logue et al., 2009). However, the precise mechanism by which GPR88 regulates the transmission of MSNs to alter behavior remains unknown. In recent years, research on MSNs subtype function has revealed that these two neuronal populations differentially regulate not only motor behaviors but also responses to rewarding and aversive stimuli (Durieux et al., 2009; Lobo et al., 2010; Kravitz et al., 2012). For instance, it has been suggested that altered D_2_R MSN transmission may disrupt inhibitory controls and avoidance in a decision conflict task (Hikida et al., 2010, 2013). Moreover, studies in humans and rodents suggest that the D_2_R modulates reward and emotional processing (Hranilovic et al., 2008; Peciña et al., 2013; Brandão et al., 2015), while the activation of D_2_R neurons in mice induced depressive-like behavior (Francis et al., 2015).

To gain a better understanding of how GPR88 in D_2_R MSNs regulates emotional processing, we generated a conditional knockout (KO) of *Gpr88* in neurons expressing A_2A_R (*A_2A_R*-*Gpr88* KO) known to be selectively expressed in D_2_R MSNs (Schiffmann et al., 1991; Schiffmann and Vanderhaeghen, 1993). To evaluate the contribution of GPR88 in D_2_R MSNs, we compared behavioral responses of *A_2A_R*-*Gpr88* KO mice with those of total *Gpr88* KO animals using behavioral tests measuring anxiety-like behaviors and fear responses. We show that *Gpr88* expressed in these neurons is responsible for ethological anxiety-like behaviors, but does not regulate conflict anxiety and fear responses.

## Materials and Methods

### Subjects

Mice (male and female) 9–15 weeks of age were bred in-house in a group house of three to five animals per cage. Animals where maintained on a 12 h light/dark cycle at a controlled temperature (22 ± 1°C). Food and water were available *ad libitum* throughout all experiments except for the novelty suppressed feeding test. All experiments where approved by the local ethics committee (COMETH 2014-029). For all experiments, *Gpr88* *^A2A-Cre^* mice were compared with their littermates (*Gpr88^flx/flx^*), and *Gpr88* ^−/−^ mice were compared with *Gpr88^+/+^* mice. An independent cohort of naive animals was used for each behavioral paradigm, except for the fear conditioning that was performed in the same cohort as the light/dark test 48 h after the latter. All behavioral testing was performed and analyzed blind to genotypes.

### Generation of *Gpr88* ^−/−^ and *Gpr88* ^A2A-Cre^ mice

*Gpr88*-floxed mice (*Gpr88^flx/flx^*), total *Gpr88* KO mice (*Gpr88* ^−/−^; Meirsman et al., 2016), and *Adora2a*-*Cre* mice (Durieux et al., 2009) were produced as previously described. Briefly, to generate a total KO, *Gpr88^flx/flx^* mice, in which exon 2 is flanked by a loxP site (upstream) and a Lox-FRT neomycin-resistance cassette (downstream) were crossed with cytomegalovirus (CMV)-Cre mice expressing Cre recombinase under the cytomegalovirus promoter. This led to germline deletion of *Gpr88* exon 2 under a mixed background (13.96%, C57BL/6; 60.94%, C57BL/6J; 0.05%, FVB/N; 25%, 129/SvPas; 0.05%, SJL/J).

To generate a conditional KO of *Gpr88* in A_2A_R-expressing neurons (*Gpr88* *^A2A-Cre^*), *Adora2a-Cre* mice were crossed with *Gpr88^flx/flx^* mice. The introduction of loxP sites in the mouse *Gpr88* gene had no effect on the agonist-induced activation of GPR88 receptor in homozygous floxed mice (*Gpr88^flx/flx^*) compared with wild-type animals (*Gpr88^+/+^*; see Fig. 2*A*). First-generation animals expressing Cre under the control of A_2A_R promotor (Gpr88*^A2A-Cre/+^*) were crossed a second time to eliminate the wild-type *Gpr88* gene (*Gpr88* *^A2A-Cre^*; background: 1.08%, C57BL/6; 16.78%, C57BL/6J; 0.01%, FVB/N; 53.17%, 129/SvPas; 0.01%, SJL/J; 29.54%, C57BL/6N). All mice were bred at the Institut Clinique de la Souris-Institut de Génétique et Biologie Moléculaire et Cellulaire.

Mice were genotyped using PCR-based genotyping with the following primers: *Gpr88* ^−/−^ mice, 5'-GAAGAGTGA AACCACAGGTGTGTACAC-3' and 5'-GTT TGT TTC CTC ACT GGC TGA GAG TC-3' for GPR88^+/+^; and *Gpr88^A2A-Cre^* mice, 5'-GTC CTA GGT GTG GAT ATG ACC TTA G-3' and 5'-GTT TGT TTC CTC ACT GGC TGA GAG TC 3'. To verify the presence of Cre and myosine (with the latter as a positive control) the following primers were used: 5'-GAT CGC TGC CAG GAT ATA CG-3', 5'-CAT CGC CAT CTT CCA GCA G-3', 5'-TTA CGT CCA TCG TGG ACA GC-3', and 5'-TGG GCT GGG TGT TAG CCT TA-3'.

### Tissue preparation and fluorescent *in situ* hybridization

Mice (*n* = 4 *Gpr88^flx/flx^;* 4 *Gpr88* *^A2A-Cre^*) were killed by cervical dislocation, and fresh brains were extracted and embedded in OCT (Optimal Cutting Temperature Medium, Thermo Scientific), frozen, and kept at −80°C. Frozen brains were coronally sliced into 20 µm serial sections by using a cryostat (model CM3050, Leica) and placed on SuperFrost slides (Thermo Scientific). *In situ* hybridizations were performed using the RNAscope Multiplex Fluorescent Assay. GPR88 probes were coupled to FITC, while D_1_R and D_2_R probes were coupled with Tritc and Cy5, respectively.

### Relative expression of *Gpr88* mRNA in *Drd1*- and *Drd2*-positive cells

**Figure 1.**
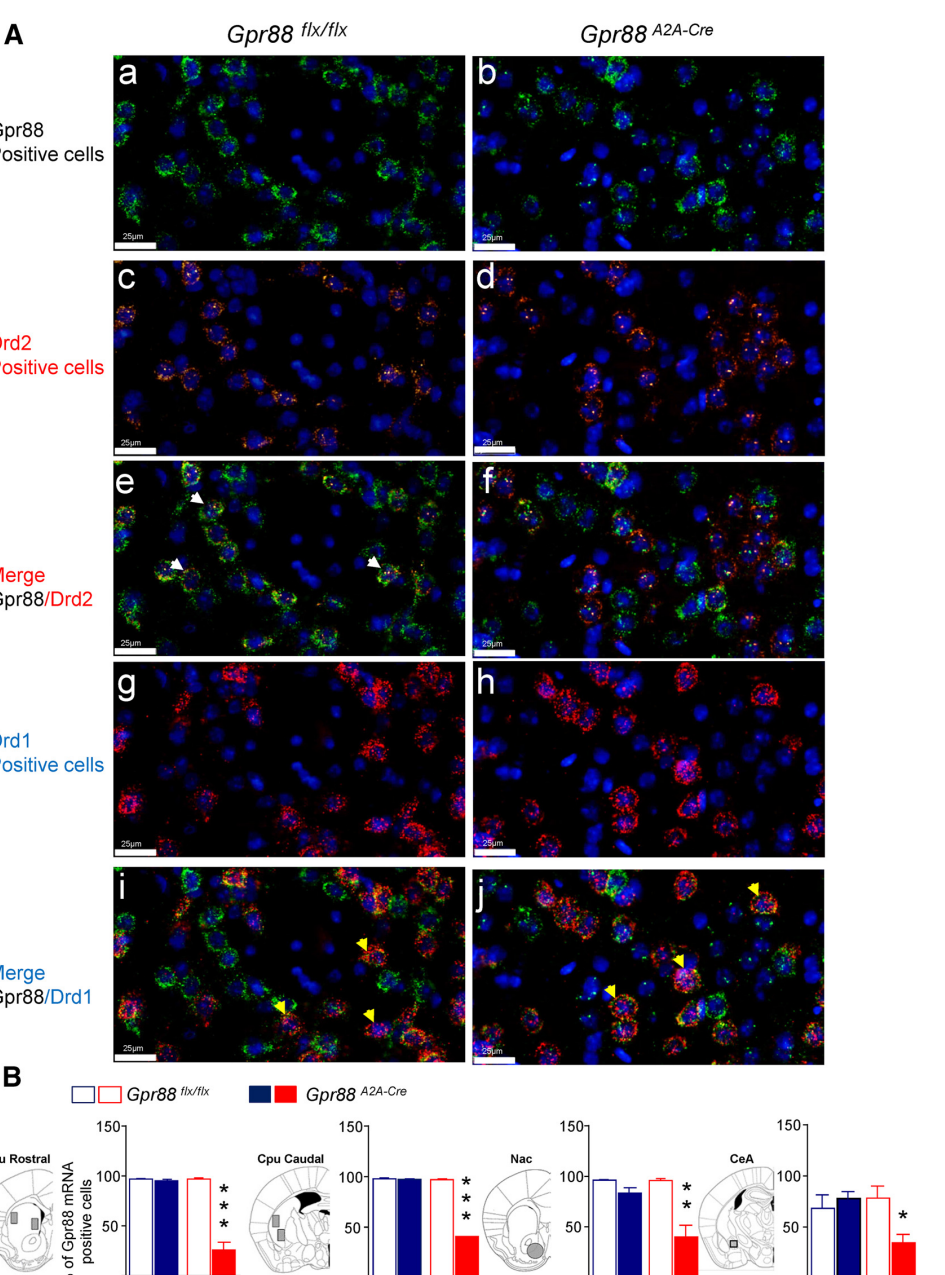
Molecular characterization of conditional *A_2A_R*-*Gpr88* KO mice. ***A***, Triple-fluorescent *in situ* hybridization probing Gpr88 (***Aa***, ***Ab***, ***Ae***, ***Af***, ***Ai***, and ***Aj***; probe labeled in green), Drd2 (***Ac–Af***; probe labeled in orange), and Drd1 (***Ag–Aj***; probe labeled in red). Representative images are shown. In *Gpr88*^flx/flx^ control animals, Gpr88 mRNA colocalizes with both Drd2 (***Ae***: merge GPR88/Drd2, white arrows) and Drd1 mRNA (***Ai***: merge GPR88/Drd1, yellow arrows). In contrast, *Gpr88A2A-Cre* conditional mice show almost no colocalization with Drd2 (***Af***: merge GPR88/D_2_R), while colocalization with Drd1 remains (***Aj***: merge GPR88/Drd1, yellow arrows). DAPI staining (blue) was used to label all cell nuclei. Scale bar, 25 µm. ***B***, Quantification shows a strong decrease of Gpr88/Drd2 double-positive neurons (red), but not GPR88/Drd1 double-positive (blue) neurons, in the CPu, Nac, and CeA of *Gpr88A2A-Cre* conditional mice. Percentage of Gpr88 expression was calculated based on the total number of Drd1- or Drd2-positive cells counted [(number Drd1- or Drd2-expressing cells coexpressing Gpr88 × 100)/total number of Drd1- or Drd2-expressing cells]. Data are presented as the mean ± SEM. *n* = 4, Gpr88A2A-Cre; *n* = 4, Gpr88^flx/flx^; solid asterisks). *p < 0.05; **p < 0.01; ***p < 0.001 (Student’s *t* test).

Image acquisition was performed with the slide scanner NanoZoomer 2 HT and fluorescence module L11600-21 (Hamamatsu Photonics). To verify the specific excision of *Gpr88* in D_2_R MSNs neurons, *in situ* hybridization images were analyzed using NDP.viewer software. For each brain, four slices were selected, as follows: two slices for the caudate–putamen (CPu; rostral, +0.98 mm from bregma; caudal, −0.58 mm from bregma); one slice for the nucleus accumbens (Nac; +1.34 mm relative to bregma); and one slice for the central nucleus of the amygdala (CeA; −1.22 from bregma). For each structure, regions of interest (ROIs) were determined by drawing two*-*dimensional boxes with defined surfaces. Counting was performed on one ROI with a surface of 1 mm^2^ for the Nac, 0.250 mm^2^ for the CeA, and two ROIs of 0.5 mm^2^ for the each CPu slice (to include both dorsomedian and dorsolateral striatum; Fig. 1). Counting was balanced between right and left hemispheres. To evaluate the expression of *Gpr88* mRNA in D_1_R- and D_2_R-expressing cells, counting was performed manually using the NDP.view software counting add-up. First, cells expressing *Drd1* mRNA, but not *Drd2* mRNA, were marked and counted. For each *Drd1*-positive cell, the coexpression of *Gpr88* was verified and counted separately. This process was repeated for *Drd2* mRNA-positive cells. Relative *Gpr88* expression is represented as a percentage of the total *Drd1*- or *Drd2*-positive cells counted [(number *Drd1* or *Drd2* expressing cells coexpressing *Gpr88* × 100)/total number of *Drd1*- or *Drd2*-expressing cells]. Given the lack of difference in *Gpr88* expression between lateral and medial CPu, the relative percentage of each was pooled for graphical representation and statistical analysis.

### [^35^S]-GTPγS binding assay

[^35^S]-GTPγS assays were performed on membrane preparations, as described in our previous report (Pradhan et al., 2009).

To evaluate the activation of GPR88 in PFC, CPu, Nac, CeA, and hippocampus, structures were punched in six animals of each genotype (three males, three females), as previously described (Meirsman et al., 2016), and were pooled for membrane preparation. To perform [^35^S]-GTPγS assays on whole-striatum mice were killed by cervical dislocation, and both striata were rapidly manually removed, frozen in dry ice, and stored at −80°C until use. Three membrane preparations were used for each genotype, gathering tissue from three animals each (males and females). Results are expressed as mean measures from the three membrane preparations. All assays were performed on membrane preparations. Membranes were prepared by homogenizing the tissue in an ice-cold 0.25 m sucrose solution (10 vol/ml/g wet weight of tissue). Samples were then centrifuged at 2500 × *g* for 10 min. Supernatants were collected and diluted 10 times in buffer containing 50 mm Tris-HCl, pH 7.4, 3 mm MgCl_2_, 100 mm NaCl, and 0.2 mm EGTA, following which they were centrifuged at 23,000 × *g* for 30 min. The pellets were homogenized in 800 μl of ice-cold sucrose solution (0.32 m) and kept at −80°C. For each [3^5S^]GTPγS binding assay, 2 μg of protein/well was used. Samples were incubated with and without ligands, for 1 h at 25°C in assay buffer containing 30 mm GDP and 0.1 nm [^35^S]GTPγS. Bound radioactivity was quantified using a liquid scintillation counter. *B*_max_ and *K*_d_ values were calculated. Nonspecific binding was defined as binding in the presence of 10 μm GTPγS, and binding in the absence of agonist was defined as the basal binding.

### Drugs

The GPR88 agonist compound 19 (Meirsman et al., 2016) was synthesized by Prestwick Chemicals and dissolved in water.

### Behavioral analysis

#### Open field locomotion

To assess basal locomotor activity in a novel environment, mice were placed in a dimly lit (15 lux) open field arena placed over a white Plexiglas infrared-lit platform. Locomotor activity was recorded during 30 min via an automated tracking system (Videotrack, ViewPoint). Only movements with speed exceeding 6 cm/s were taken into account for this measure.

#### Elevated plus maze

Anxiety-like behavior was first evaluated using the ethological (also known as unconditioned) anxiety test elevated plus maze (EPM). The EPM was a plus-shaped maze elevated 52 cm from the base, with a black Plexiglas floor consisting of two open and two closed arms (37 × 6 cm each) connected by a central platform (6 × 6 cm). The experiments were conducted under low-intensity light (15 lux). The movement and location of the mice were analyzed by an automated tracking system (Videotrack; View Point, Lyon, France). Each mouse was placed on the central platform facing a closed arm and were observed for 5 min. Anxiety-like behavior was assessed by measures of the time spent and the number of entries in closed and open arms of the maze, and related time and activity ratios (time spent or distance traveled in open arms/total time spent or distance in arms). Risk-taking behavior was evaluated by analyzing the time spent in the distal part of the open arms (time spent in the last one-third of the open arm) and the number of head dips (total number of head dips and head dips from the distal part of the open arms). Finally, the distance traveled in the maze was used as a measure of locomotor activity.

#### Light/dark test

Anxiety-like behavior was next evaluated using the light/dark apparatus, which was composed of two rectangular compartments (20 × 20 × 14 cm) separated by a tunnel (5 × 7 × 10 cm; Imetronic). One compartment is constituted of a black floor and walls dimly lit (5 lux), whereas the other is constituted of a white floor and walls intensely lit (1000 lux). The apparatus is equipped with infrared beams and sensors. Mice were placed in the dark compartment, and behavior was automatically recorded for 5 min.

#### Novelty-suppressed feeding test

Conflict-based anxiety was measured using the novelty-suppressed feeding test. All mice were subjected to fasting 24 h before the beginning of the test, but water was provided *ad libitum*. Mice were isolated in a single cage 30 min before the beginning of the test. During the test, three food pellets (regular chow) were placed on a square piece of white filter paper positioned in the center of a brightly illuminated (60 lux) open field (50 × 50 cm) filled with ∼2 cm of sawdust bedding. Each mouse was placed in a corner of the open field facing the open field wall. The latency to the first bite of the food pellet was recorded. The cutoff time was defined as 15 min. After the test was over, the animal was placed in the home cage and left alone for 5 min. The food intake during this period was scored.

#### Social interaction test

Social interaction was assessed on an open field (50 × 50 cm) dimly lit (<10 lux) using naive wild-type mice of the same age and weight as interactors. On the first day, all mice were individually placed in the open field arena and left for a 10 min period of habituation. The next day, mice were placed in the open field arena with a wild-type naive interactor, and a 10 min session was recorded. Nose and paw contacts as well as following and grooming were measured. If an interactor failed to engage in any interaction, data from the respective mice were excluded from analysis (one mouse was excluded).

#### Marble burying

Defensive burying was measured using the marble-burying test performed with 20 small glass marbles (15 mm) evenly spaced in a transparent single cage (21 × 11 × 17 cm) over 4 cm sawdust bedding. The cage was covered by a plastic lid in a room illuminated at 40 lux. The mice were left in the cage for 10 min, and the number of marbles buried more than halfway in the sawdust was counted.

#### Novelty preference

Novelty preference was assessed in unbiased computerized boxes that have been previously described (Imetronic; Le Merrer et al., 2012). Briefly, the apparatus was composed of two chambers separated by a central alley. Two sliding doors separated the compartments from the central alley. Chambers differed in global shape (but same total surface) and floor texture. Mice were confined to one of the chambers (the familiar chamber) for 15 min before being placed in the central corridor for 5 min. Then, both sliding doors were opened, and mice were allowed to freely explore the apparatus. Time spent in each chamber was recorded, and the novelty preference was calculated as the percentage of time spent in the unfamiliar compartment.

#### Fear conditioning

Context- and cue-related conditioned fear responses were evaluated using a fear-conditioning paradigm. Experiments were conducted in four dimly lit operant chambers (28 × 21 × 22 cm; Coulbourn Instruments), with a Plexiglas door and a metal bar floor connected to a shocker (Coulbourn Instruments). Chambers had a permanent house light and were equipped with a speaker for tone delivery. An infrared activity monitor, which was used to assess animal motion, was placed on the ceiling of each chamber. The activity/inactivity behavior was monitored continuously during a 100 ms period. Data are expressed in the duration of inactivity per second, and the total time of inactivity displayed by each subject during training and testing sessions was counted. The procedure was similar to one previously described (Goeldner et al., 2009). Briefly, on the first day, animals underwent one conditioning session; on the second day, contextual and cued fear conditioning were tested. The conditioning session was initiated with a 4 min habituation period followed by a 20-s-long tone of 20 KHz/75 dB [conditional stimulus (CS)] coupled with a 0.4 mA footshock [unconditional stimulus (US)] during the last second. Two minutes later, a similar CS–US pairing was presented, and the mice were removed from the apparatus 2 min after the footshock. On the following day, mice were exposed again to the conditioning chamber, and immobility was measured over 4 min to assess contextual fear conditioning. The same day, 5 h after context fear was measured, cued fear conditioning was assessed in modified chambers.

### Statistical analysis

All data are expressed as the mean group value ± SEM and were analyzed using Student’s test or two-way ANOVA, whenever it was appropriate. When relevant, data were submitted to Sidak’s or Tukey’s multiple-comparison *post hoc* analysis. The criterion for statistical significance was *p* < 0.05. All statistics were performed using Prism 6 (GraphPad Software).

## Results

### *Gpr88^A2A-Cre^* mice show decreased *Gpr88* mRNA levels in D_2_R neurons of caudate–putamen, nucleus accumbens, and central amygdala

To conditionally delete *Gpr88* exon 2 in D_2_R MSNs, we crossed mice carrying two LoxP sites flanking the second exon of the *Gpr88* gene with mice expressing the Cre recombinase under the control of the D_2_R MSN-specific *Adora2a* gene promoter (Durieux et al., 2009; Meirsman et al., 2016). Then, we quantified *Gpr88* mRNA in *Drd1*-positive and *Drd2*-positive neurons in *Gpr88^flx/flx^* (control) and *Gpr88^A2A-Cre^* mice using triple *in situ* hybridization. Quantitative analysis was performed in four brain regions, including rostral CPu, caudal CPu, Nac, and CeA (Fig. 1). In control animals (*n* = 4), *Gpr88* mRNA was present in striatal *Drd1* (96.76%±0.28) and *Drd2* (96.44%±0.74) expressing cells, as well as in the few (*n* ∼ 17/ROI) cells coexpressing the two dopaminergic receptor mRNAs. In the CeA of control animals, *Gpr88* was expressed in fewer *Drd1*-positive (68.31%±13.24) and *Drd2*-positive (78.26%±11.77) cells compared to the striatum, and did not significantly differ across the two cell types (*t*_(6)_=0.56; *p* = 0.59). Expression of the Cre in A_2A_R expressing neurons had no effect on *Gpr88* mRNA in D_1_R expressing neurons in any of the structures analyzed (*n* = 4). In contrast, the number of *Gpr88-*positive cells was strongly reduced in D2R expressing MSNs of rostral ((25.89%±7.86); *t*_(6)_ =9.004; *p* < 0.001) and caudal CPu (40.24 ± 2.68%; *t*_(6)_ =19.68; *p* < 0.0001) as well as in Nac (40.08 ± 11.66%; *t*_(6)_=4.736; *p* = 0.0032) as previously demonstrated with this *Adora2a* cre mice (Durieux et al., 2009, 2012; Ena et al., 2013). Also, mice expressing the Cre in A_2A_R expressing neurons had significantly lower number of *Gpr88*-positive cells in D_2_R-neurons of the CeA (34.33%±8.2) compared to control animals (*t*_(6)_ =3.06; *p* = 0.02). Together, the data indicate that conditional *Gpr88^A2A-Cre^* mice show a selective decrease of *Gpr88* transcript levels in D_2_R-neurons of striatum and CeA.

### *Gpr88^A2A-Cre^* mice show decreased GPR88 agonist-induced [S35]-GTPγS binding

**Figure 2.**
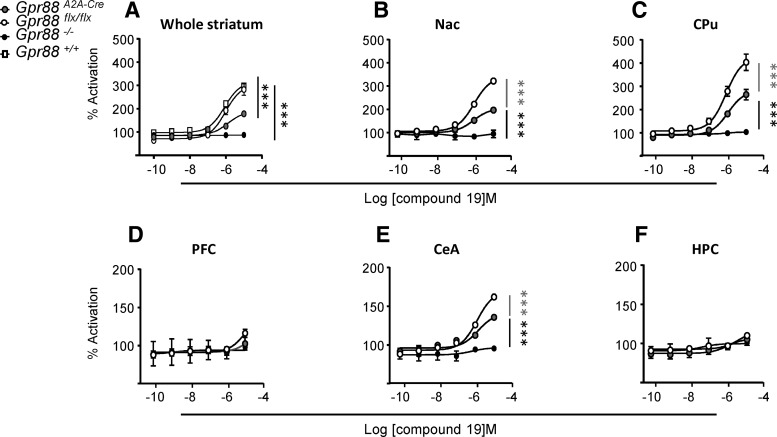
Agonist-induced GPR88 activation in conditional *A_2A_R*-*Gpr88* KO mice. Introduction of LoxP sites does not alter GPR88 activation. ***A***, GPR88-mediated [^35^S]-GTPγS was totally and partially abolished in the striatum of *Gpr88* ^−/−^ and *Gpr88^A2A-Cre^* mice, respectively. *Gpr88^+/+^* and *Gpr88^flx/flx^* mice present similar GPR88 agonist-induced receptor activation. Two membrane preparations were used per genotype, with each membrane preparation gathering tissue from three animals (1 male/2 females or 2 males/1 female). ***B–F***, Decreased activation of GPR88 in several brain regions of *Gpr88^A2A-Cre^* mice: GPR88-mediated [^35^S]-GTPγS binding is decreased in the CPu, Nac, and central amygdala of mutant animals. Data are represented as the mean ± SEM. ***A***, Text: ***p < 0.001 (*post hoc* Tukey’s multiple-comparisons test of *Gpr88^flx/flx^* or Gpr*88^+/+^* vs *Gpr88^A2A-Cre^* and *Gpr88* ^−/−^; ***B–F***); *n* = 6, *Gpr88^A2A-Cre^*; *n* = 6, *Gpr88* ^−/−^; *n* = 6, *Gpr88^flx/flx^*; text: ***p < 0.001 (*post hoc* Tukey's multiple comparisons test).

To measure the consequences of *Gpr88* gene knockout at protein level, we performed GPR88 agonist-induced [^35^S]-GTPγS binding assays in *Gpr88^A2A-Cr^* ^e^ mice and their controls, as well as in total *Gpr88* ^−/−^ (negative control; Fig. 2*B–F*). Structures were chosen based on A_2A_R expression (Schiffmann et al., 1991; Schiffmann and Vanderhaeghen, 1993). We found a significant genotype effect in the CPu (*Gpr88^A2A-Cre^*: 282.2%±15.46; *Gpr88^flx/flx^*: 427%±22.61; genotype effect *F*_(2,30)_=61.56; *p* < 0.0001), Nac (*Gpr88^A2A-Cre^*: 205.1%±5.54; *Gpr88^flx/flx^*: 342.4%±9.13; *F*_(2,30)_=152.9; *p* < 0.0001), and CeA (*Gpr88^A2A-Cre^*: 141.2%±6.18; *Gpr88^flx/flx^*: 170.3%±5.90; *F*_(2,30)_=37.48; *p* < 0.0001). *Post hoc* analysis (Tukey multiple comparisons) revealed significant differences between *Gpr88* *^A2A-Cre^* and *Gpr88* *^flx/flx^* for the two highest agonist concentrations (10^−5^ and 10^−6^ M) in the CPu, Nac, and CeA. This result demonstrates that the selective *Gpr88* gene KO in D_2_R-expressing cells, observed at mRNA level, translates into a significant reduction of protein levels in regions of high *Gpr88* expression. Although the approach does not discriminate GPR88 signaling in D_1_R and D_2_R-expressing cells, the approximately 40% reduction in CPu membranes reflects the specific *Gpr88* gene KO in D_2_R cells, representing ∼40% of the dorsal striatum population of dopamine receptor-expressing neurons (Valjent et al., 2009)

### Both total and A_2A_R-*Gpr88* gene deletion increase basal locomotor activity

**Figure 3.**
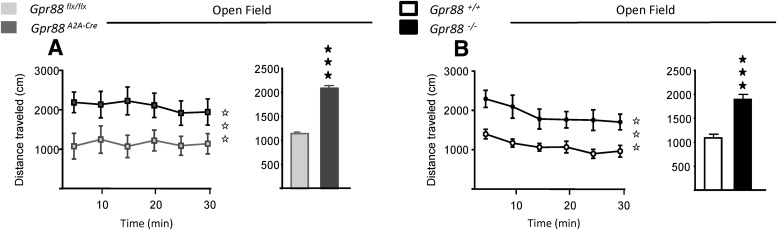
Locomotor activity is similarly increased in *A2AR*-*Gpr88* KO and total KO mice. ***A***, ***B***, When placed individually in a dimly lit open field for 30 min, both *A2AR*-*Gpr88* KO mice (***A***) and total KO mice (***B***) traveled a longer distance than their control littermates. Line graphs show the distance traveled (in centimeters) in 5 min bins over a 30 min session. Bar graphs show the average total distance traveled (in centimeters) over the 30 min sessions. Data are represented as the mean ± SEM. *n* = 10, *Gpr88^A2A-Cre^*; *n* = 10, *Gpr88^flx/flx^* (***A***); *n* = 12, Gpr88^−/−^; *n* = 12, *Gpr88^+/+^* (***B***). Open asterisks: ***p <0.001 (repeated-measures ANOVA); solid asterisks: ***p < 0.001 (Student’s *t* test).

Previous studies have demonstrated increased basal locomotor activity in total *Gpr88* KO mice (Quintana et al., 2012; Meirsman et al., 2016), as was also observed in mice with disrupted D_2_R MSN activities (Durieux et al., 2009, 2012; Bateup et al., 2010). We compared general locomotor activity in total and *A_2A_R*-*Gpr88* KO mice. Animals were individually placed in a dimly lit open field and analysis of forward locomotion revealed a significant increased activity for *Gpr88^A2A-Cre^* (*n* = 10, Gpr88*^A2A-Cre^*; *n* = 10, *Gpr88^flx/flx^*; genotype effect: *F*_(1,108)_ = 28.93, *p* < 0.0001; mean locomotion: *t*_(10)_ =15.60, *p* < 0.0001) as well as *Gpr88* ^−/−^ mice (*n* = 12, Gpr88^−/−^; *n* = 12, *Gpr88^+/+^*; genotype effect: *F*_(1,132)_ = 52.72, *p* < 0.0001; mean locomotion: *t*_(10)_=6.65; *p* < 0.0001; Fig. 3). The deletion of *Gpr88* in A_2A_R-neurons is therefore sufficient to recapitulate the hyperlocomotion phenotype of *Gpr88* KO mice, suggesting that the locomotor effect of GPR88 is mediated through receptors expressed in D_2_R MSNs.

### Both total and *A_2A_R*-*Gpr88* deletion decrease anxiety-like behavior

**Figure 4.**
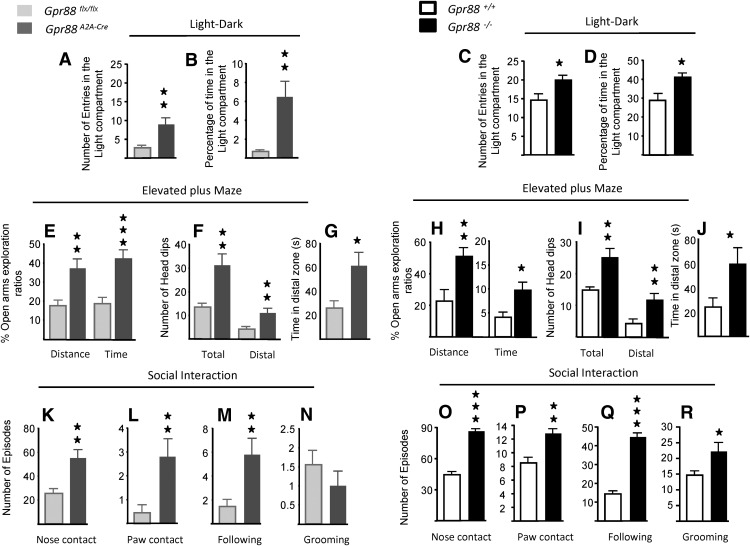
Anxiety-related responses are similarly increased in *A2AR-Gpr88* KO and total KO mice. ***A–D***, *A2AR-Gpr88* mice (***A***, ***B***) and total KO mice (***C***, ***D***) enter more frequently and spent more time in the light compartment of the light/dark test. ***E–J***, In the elevated plus maze, *Gpr88^A2A-Cre^* and *Gpr88* ^−/−^ mice present higher open arms exploration ratios (***E***, ***H***, respectively), more frequent total and distal head dips (***F***, ***I***), and increased time spent in the distal zone of the open arms when compared with their control littermates (***G***, ***J***). ***K–R***, Social interactions were evaluated in a dimly lit open field with wild-type naive mice of the same age and gender. Both mutant animals display increased number of nose and paw contacts, as well as increased following behaviors. Gpr88^−/−^ mice, but not Gpr88^A2A-Cre^ mice, engaged less frequently in grooming episodes than control animals Data are presented as the mean ± SEM. ***A***, ***B***: *n* = 11, *Gpr88^A2A-Cre^*; *n* = 9 *Gpr88^flx/flx^*; ***C***, ***D***: *n* = 10, *Gpr88* ^−/−^; *n* = 11, *Gpr88^+/+^*; ***E–G***: *n* = 12, *Gpr88^A2A-Cre^*; *n* = 13, *Gpr88^flx/flx^*; ***H–J***: *n* = 11, *Gpr88* ^−/−^; *n* = 9, *Gpr88^+/+^*; ***K–N***: *n* = 10, *Gpr88^A2A-Cre^*; *n* = 9, *Gpr88^flx/flx^*; ***O–R***: *n* = 6, *Gpr88* ^−/−^; *n* = 6, *Gpr88^+/+^*. Solid asterisks: *p < 0.05, **p < 0.01, ***p < 0.001 (Student’s *t* test).

Complete deletion of *Gpr88* in mice decreased anxiety levels in several models of anxiety-like behavior (Meirsman et al., 2016). To examine whether this behavior is dependent on GPR88 in A_2A_R neurons, we evaluated anxiety-like behaviors of *Gpr88^A2A-Cr^* ^e^ and *Gpr88* ^−/−^ mice in the standard light/dark and elevated plus maze test. Results indicate that both *A_2A_R*-*Gpr88* (*n* = 11, *Gpr88^A2A-Cre^*; *n* = 9, *Gpr88^flx/flx^*) and total KO mice (*n* = 11, *Gpr88^A2A-Cre^*; *n* = 9, *Gpr88^flx/flx^*) entered more frequently (*Gpr88^A2A-Cre^*: *t*_(18)_= 3.01, *p* = 0.008; *Gpr88* ^−/−^: *t*_(19)_= 2.46, *p* = 0.023) and spent more time (*Gpr88^A2A-Cre^*: *t*_(18)_= 3.11, *p* = 0.006; *Gpr88* ^−/−^: *t*_(19)_= 2.84, *p* = 0.01) exploring the aversive illuminated compartment of the light/dark apparatus (Fig. 4*A–D*). In the elevated plus maze, A_2A_R-*Gpr88* (*n* = 12, *Gpr88^A2A-Cre^*; *n* = 13, *Gpr88^flx/flx^*) and total *Gpr88* deletion (*n* = 11, *Gpr88* ^−/−^; *n* = 9, *Gpr88^+/^* ^+^) increased the open arms (OA) exploration (percentage of time in the OA: *Gpr88^A2A-Cre^*, *t*_(23)_= 4.13, *p* = 0.0004; *Gpr88* ^−/−^, *t*_(18)_= 2.71, *p* = 0.015; percentage of the distance traveled in the OA: *Gpr88^A2A-Cre^*, *t*_(23)_= 3.43, *p* = 0.0023; *Gpr88* ^−/−^, *t*_(18)_= 3.05, *p* = 0.007; Fig. 4*E*,*H*). Also, both mutants spent more time in the distal part of the open arm (*Gpr88^A2A-Cre^*, *t*_(23)_ = 2.76, *p* = 0.011; *Gpr88* ^−/−^, *t*_(18)_ = 2.17, *p* = 0.043; Fig. 4*G*,*J*) and displayed a higher number of total and distal head dips (total: *Gpr88^A2A-Cre^*, *t*_(23)_ = 3.54, *p* = 0.0017; *Gpr88* ^−/−^, *t*_(18)_ = 3.05, *p* = 0.007; distal: *Gpr88^A2A-Cre^*, *t*_(23)_ = 2.95, *p* = 0.0072; *Gpr88* ^−/−^, *t*_(18)_ = 2.81, *p* = 0.012; Fig. 4*F*,*I*). There was no difference in the total distance traveled (mutant mice, *t*_(23)_ = 0.14, *p* = 0.886; and control mice, *t*_(18)_ = 0.43, *p* = 0.675) or the total number of arms entries (mutant mice, *t*_(23)_ = 0.09, *p* = 0.925; control mice, *t*_(18)_ = 0.65, *p* = 0.52; data not shown). Together, these data show that the GPR88 deletion in A_2A_R neurons fully accounts for the high levels of anxiety observed in total GPR88 KO mice in this study and our previous study (Meirsman et al., 2016).

Because anxiety can affect social behavior, we scored for social interaction. Both mutant lines show increased nose contact (*n* = 10, *Gpr88^A2A-Cre^*; *n* = 9, *Gpr88^flx/flx^*: *t*_(17)_ = 3.39, *p* = 0.004; *n* = 6, *Gpr88* ^−/−^; *n* = 6, *Gpr88^+/+^*: *t*_(10)_ = 10.12, *p* < 0.0001) and paw contact (*t*_(17)_= 2.73, *p* = 0.014; and *t*_(10)_= 3.94, *p* = 0.0028) and followed their interactor more frequently than did control littermates (*t*_(17)_= 2.72, *p* = 0.014; and *t*_(10)_ = 12.03, *p* < 0.0001; Fig. 4*K–R*). GPR88 in A_2A_R neurons, therefore, also recapitulate the phenotype of total GPR88 KO mice in this test.

### *A_2A_R*-*Gpr88* gene deletion increases avoidance but does not regulate approach behaviors

**Figure 5.**
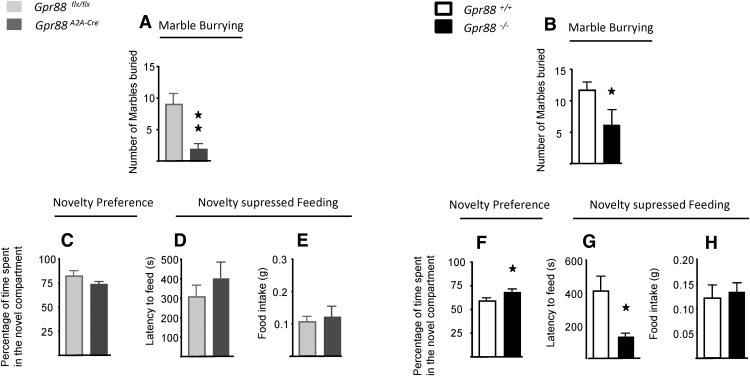
*A2AR*-*Gpr88* gene deletion increases avoidance but does not regulate approach behaviors. ***A***, ***B***, In the marble-burying test (*Gpr88^A2A-Cre^*, ***A***; for *Gpr8* ^−/−^, ***B***), both KO mice buried fewer marbles than controls. ***C***, ***F***, When assessing novelty preference, total KO mice (***F***), but not *A2AR*-*Gpr88* KO mice (***C***), spent more time in the novel compartment when compared with their littermates. ***D***, ***E***, ***G***, In the novelty-suppressed feeding test, *Gpr88^A2A-Cre^* mice exhibit similar latencies to start eating and home cage food intake (***D***) than *Gpr88^flx/flx^* mice. Gpr88^−/−^ mice display shorter latencies to start eating in the center of the arena compared with *Gpr88*^+/+^ mice (***G***), eating normally when placed back in their home cage (***E***). Data are presented as the mean ± SEM. *n* = 11, *Gpr88^A2A-Cre^*; *n* = 9, *Gpr88^flx/flx^* (***A***); *n* = 7, *Gpr88* ^−/−^; *n* = 7, *Gpr88^+/+^* (***B***); *n* = 8, *Gpr88^A2A-Cre^*; *n* = 6, *Gpr88^flx/flx^* (***C***); *n* = 11, *Gpr88^A2A-Cre^*; *n* = 11, *Gpr88^flx/flx^* (***D***, ***E***); *n* = 7, *Gpr88* ^−/−^; *n* = 7, *Gpr88^+/+^* (***F***); *n* = 7, *Gpr88* ^−/−^; *n* = 7, *Gpr88^+/+^* (***G***, ***H***). Solid asterisk: *p < 0.05, **p < 0.01 (Student’s *t* test).

Altered anxiety can be explained by an imbalance between approach-related and avoidance-related drives (Aupperle and Paulus, 2010). To further investigate the role of GPR88 in approach–avoidance conflict-based anxiety tests, we measured avoidance and approach behaviors separately in both mouse lines. Marble burying constitutes an unconditioned defensive behavior aiming to avoid threats, and is reversed by anxiolytic drugs (De Boer and Koolhaas, 2003). In the presence of 20 marbles, *A_2A_R*-*Gpr88* mice (*n* = 7, *Gpr88^A2A-Cre^*; *n* = 9 *Gpr88^flx/flx^*: *t*_(14)_ =3.28, *p* = 0.005) and total KO mice (*n* = 7, *Gpr88* ^−/−^; *n* = 7, *Gpr88^+/+^*: *t*_(12)_ =2.42, *p* = 0.032) buried significantly fewer marbles then their control littermates (Fig. 5*A*,*B*). In this test, therefore, conditional D_2_R mice recapitulate the behavioral deficit of total KO mice. However, when evaluating the motivation of mice for the exploration of novel instead of familiar environments (Fig. 5*C*,*F*), we observed a significant increase in novel environment exploration for *Gpr88* ^−/−^ mice (*n* = 7, *Gpr88* ^−/−^; *n* = 7 *Gpr88^+/+^*: *t*_(12)_ =2.31, *p* = 0.039), but not for *Gpr88^A2A-Cre^* mice (*n* = 7, *Gpr88^A2A-Cre^*; *n* = 9, *Gpr88^flx/flx^*: *t*_(12)_ =1.43, *p* = 0.17). Moreover, in the novelty-suppressed feeding test (Fig. 5*D*,*G*) *Gpr88* ^−/−^ mice, but not *Gpr88^A2A-Cre^* mice, show a decreased latency to feed. Thus, approach behaviors in both novelty preference and novelty-suppressed feeding tests are enhanced in total but not in *A_2A_R GPR88* KO mice. These data together suggest that GPR88 in A_2A_R neurons regulates threat avoidance, but not novelty approach behaviors.

### Total but not *A_2A_R*-*Gpr88* deletion impairs fear conditioning

**Figure 6.**
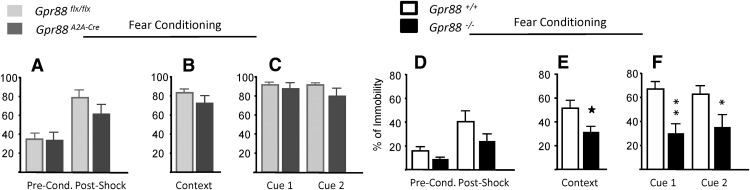
Total but not *A2AR*-*Gpr88* gene deletion impairs fear conditioning. To assess whether *Gpr88* deletion affects fear responses, we tested mice in a fear-conditioning test. ***A***, ***D***, During the conditioning session, mutant and control animals displayed similar levels of immobility before and after tone–shock pairing when compared with control mice. ***E***, Twenty-four hours later, *Gpr88* ^−/−^ mice displayed significantly lower context fear than *Gpr88^+/+^* mice. ***F***, The percentage of immobility of *Gpr88* ^−/−^ was also decreased when tested for cued fear memory. Deletion of *Gpr88* in A_2A_R-expressing neurons did not affect context (***B***) or cued (***C***) fear memories. *n* = 11, *Gpr88^A2A-Cre^*; *n* = 11, *Gpr88^flx/flx^* (***A–C***); *n* = 10, *Gpr88* ^−/−^; *n* = 10, *Gpr88^+/+^* (***D–F***). Solid asterisks: *p < 0.05 (Student’s *t* test); text asterisks: *p < 0.05, **p < 0.01 (*post hoc* Sidak’s multiple comparisons test).

Fear and anxiety are related processes, and fear circuitry has been shown to be altered in anxiety disorders (Stein et al., 2007). To evaluate whether *Gpr88* deletion affects fear learning and expression, we tested mice in a fear-conditioning paradigm (Fig. 6). During conditioning, postshock immobility increased regardless of the genotype, which is suggestive of successful US–CS conditioning (*n* = 11, *Gpr88^A2A-Cre^*; *n* = 11, *Gpr88^flx/flx^*: *F*_(1,20)_ =23.25, *p* = 0.0001; *n* = 10, *Gpr88* ^−/−^; *n* = 10, *Gpr88^+/+^*: *F*_(1,18)_ =17.05, *p* = 0.0006; time-effect repeated-measures ANOVA). When tested for contextual fear, *Gpr88* ^−/−^ mice displayed significantly less immobility than control animals (*t*_(18)_ =2.36, *p* = 0.029), which is in agreement with the altered hippocampal functioning previously reported in *Gpr88* KO mice (Meirsman et al., 2016). A similar profile was found when mice were presented with a US-paired tone. In fact, ANOVA indicates a significant genotype effect for *Gpr88* ^−/−^ cue testing (*F*_(1,18)_ =8.89, *p* = 0.008), and *post hoc* analysis (Sidak’s multiple-comparisons test) indicates a significant immobility decrease for both cues presented. In *Gpr88^A2A-Cre^* mice, context-related immobility scores were similar to *Gpr88^flx/flx^* control mice (*t*_(20)_ =1.14, *p* = 0.27). As in context fear measurement, cue presentation led to similar immobility scores between *Gpr88^A2A-Cre^* mice and their controls (*F*_(1,20)_ = 1.06, *p* = 0.31). Overall, these data show that complete deletion of *Gpr88* impairs fear expression in mice; however, deletion of this gene in A_2A_R neurons does not affect fear expression.

## Discussion

In the present study, we show that GPR88 in A_2A_R-expressing neurons regulates anxiety-like behaviors without affecting fear conditioning and drive toward novelty or food. *In situ* hybridization and GTPγS binding data indicate that *Gpr88* was efficiently deleted in neurons expressing the D_2_R of *Gpr88^A2A-Cre^* animals in dorsal as well as ventral striatum, and, to a smaller extent, in the central nucleus of the amygdala. Importantly, *Gpr88* mRNA level in D_1_R-MSNs was intact in *Gpr88^A2A-Cre^* mice when compared with control mice not expressing the Cre recombinase. The selective deletion of *Gpr88* in D_2_R neurons in the striatum of *Gpr88^A2A-Cre^* mice is consistent with the previous characterization of *Adora2a*-Cre mice showing specific expression of Cre recombinase in these neurons (Durieux et al., 2009, 2012; Ena et al., 2013). Furthermore, we confirm previous data indicating that GPR88 is present in the vast majority of D_1_R- and D_2_R-expressing MSNs (Quintana et al., 2012).

Converging evidence supports the inhibitory function of striatal D_2_R MSNs in motor output systems. Optogenetic bilateral excitation of these neurons was shown to decrease the initiation of locomotor activity (Kravitz et al., 2010), while ablation or disruption of these neurons increased motor activity (Durieux et al., 2009, 2012; Bateup et al., 2010). Several reports (Logue et al., 2009; Quintana et al., 2012), including our own work (Meirsman et al., 2016), indicate that *Gpr88* gene deletion leads to general hyperactive behavior. In the present report, we show that the deletion of *Gpr88* from A_2A_R neurons is sufficient to increase locomotor activity. Considering the predominant expression of A_2A_Rs in D_2_R MSNs, this observation suggests that GPR88 normally promotes the demonstrated inhibitory function of D_2_R MSNs on locomotor activity. The mechanisms underlying the positive modulatory role of GPR88 on D_2_R MSNs remain to be clarified. Importantly, both total and *A_2A_R*-*Gpr88* KO mice show a similar hyperactivity when tested under the same experimental conditions, strongly suggesting that the presence of GPR88 in D_2_R MSN fully accounts for this phenotype.

In a previous report, we showed that *Gpr88* ^−/−^ mice present decreased anxiety-like behaviors (Meirsman et al., 2016). Also, recent reports suggest that D_2_Rs and D_2_R MSNs regulate emotional processing and goal-directed behavior (Hranilovic et al., 2008; Kravitz et al., 2012; Peciña et al., 2013; Brandão et al., 2015; Francis et al., 2015). In the light/dark and elevated plus maze tests, both *Gpr88^A2A-Cre^* and *Gpr88* ^−/−^ mice displayed similar decreased anxiety-like behaviors with increased exploration of the light compartment/open arm of the apparatus. This strongly suggests that GPR88 in D_2_R neurons, but not in D_1_R neurons, regulate anxiety-like behaviors; however, we cannot exclude that GPR88 also regulates emotional behavior in A2AR-expressing neurons at extrastriatal sites (Wei et al., 2014). Note that this anxiety phenotype cannot be explained by their overall hyperactive behavior since the total distance traveled or the number of entries did not differ from that of control animals.

In these ethological anxiety tests, the tendency to avoid threatening stimuli (bright light/exposed arms) is confronted with the inner drive toward exploration, and this conflict is thought to inhibit exploration (Crawley, 2000; Sousa et al., 2006; Bailey and Crawley, 2009; Aupperle and Paulus, 2010). As such, the low-anxiety phenotype of mice lacking GPR88 could result from increased drive toward novelty exploration, decreased avoidance of a threatening environment, or both factors. We therefore evaluated avoidance behavior in the marble-burying test that measures ethological defensive burying (Borsini et al., 2002; De Boer and Koolhaas, 2003). Both *Gpr88* ^−/−^ and *Gpr88^A2A-Cre^* mice buried fewer marbles than control littermates, showing decreased defensive burying that is consistent with reduced threat avoidance in these mice. To tackle approach behavior, we assessed novelty preference in both KO lines. Total but not *A_2A_R*-*Gpr88* KO mice showed enhanced preference for the novel compartment when presented with a choice for novel or familiar environment. Similarly, in the novelty-suppressed feeding test, total but not *A_2A_R*-*Gpr88* deletion decreased the latency to start eating. In this conflict test, both approach and avoidance components are enhanced by starving and neophagia, respectively. The absence of phenotype of *Gpr88^A2A-Cre^* mice in these two tests could therefore be explained by unaltered motivation toward new environment or food reinforcement.

Recent reports suggest that D_1_R MSNs encode predictive reward and mediate approach behavior, while D_2_R MSNs mediate aversive and defensive behavior (Hranilovic et al., 2008; Durieux et al., 2009; Hikida et al., 2010, 2013; Kravitz and Kreitzer, 2012; Kravitz et al., 2012, 2013; Kravitz and Bonci, 2013; Calabresi et al., 2014). For instance, D_2_R MSN neurotransmission blocking in the nucleus accumbens was found to disrupt aversive but not reward learning (Hikida et al., 2010). Moreover, the same authors showed that the impaired aversive behavior was dependent on D_2_R activation (Hikida et al., 2013). Interestingly, in the present report, we show decreased threat avoidance in mice lacking GPR88 in A_2A_R neurons. Together with the increased locomotion observed in A_2A_R-*Gpr88* KO mice, we may therefore hypothesize that the lack of GPR88 in D_2_R MSNs disrupted the activity of this pathway. Further studies using electrophysiological approaches would help to confirm this hypothesis. Also, results from *Gpr88* ^−/−^ mice showing both decreased avoidance behavior and increased novelty and food approach suggest that GPR88 in D_1_R MSNs normally regulates approach behavior.

Finally, we tested the fear responses of total and *A_2A_R*-*Gpr88* KO mice for the first time. Total deletion of *Gpr88* impaired both context and cued fear responses. Reduced fear responses in total KO mice is in agreement with altered cue-based learning previously reported in *Gpr88* KO animals. The lack of phenotype in *Gpr88^A2A-Cre^* mice may be due to the D_1_R neuron-mediated mechanisms contributing to these behaviors. Alternatively, and because central amygdala functioning is essential in the acquisition and expression of fear conditioning (Wilensky et al., 2006), the partial *Gpr88* deletion at the level of the amygdala may be insufficient to alter fear responses. Further studies using viral approaches will define the precise role of GPR88 function in amygdala-mediated fear responses.

In sum, our analysis of *Gpr88^A2A-Cre^* mice shows that GPR88 in A_2A_R MSNs regulates locomotor and anxiety behaviors. These results represent a first step toward understanding the circuit mechanisms underlying GPR88 function in the brain. Future studies will evaluate the role of GPR88 in D_1_R MSNs, and how this receptor regulates the D_1_R/D_2_R MSN balance. Finally, further demonstration of GPR88 implication in anxiety-related behaviors and threat evaluation definitely posit GPR88 blockade as a new target for the treatment of anxiety-related disorders (Aupperle and Paulus, 2010).

## Synthesis

The decision was a result of the Reviewing Editor Trevor Drew and the peer reviewers coming together and discussing their recommendations until a consensus was reached. A fact-based synthesis statement explaining their decision and outlining what is needed to prepare a revision is listed below. The following reviewers agreed to reveal their identity: John Kalaska, Denise Henriques

The authors studied the learning rates of 4 groups of subjects that performed reaching movements in different ranges of external skew-viscous force fields (FFs) and hand/wrist configurations. They found that the "Control" group that made reaching movements in only one FF with one arm configuration learned very rapidly. A second group ("Cartesian") that performed reaching movements in the same FF as the control subjects (i.e., fixed in Cartesian space) learned almost as well as the control group despite experiencing the FF while holding their hand in one of three wrist postures. The third group ("Object") also learned to compensate for FFs that rotated with the wrist posture, as if the FFs were coupled to the spatial orientation of a fictive object held in their hand, except that the learning rate was slower than for the first two groups. The fourth group ("Anti-object") encountered FFs that rotated in the direction opposite to the posture of the hand and showed only slow, partial compensation for the fields.

These data are interesting and are a useful contribution to the field. However, I have to question whether they are as novel and insightful as the authors claim in the manuscript.

First, the authors should provide a clearer explanation of how these new results provide a better insight into the coordinate framework(s) for motor learning than their earlier study that used generalization to try to assess frameworks for learning (Berniker et al 2014). I think I know, but they do not express it explicitly enough. They should also provide a clearer explanation of what they mean by the title of the manuscript. What does "sculpt behaviorally relevant representations for motor learning" mean and how does that advance our understanding of motor learning? They use the word "sculpting" a number of other times in the text. How does that differ from the conclusion in Berniker et al 2014 that subjects may use a task-specific mixture of frameworks to learn a particular motor skill? Isn't that also "sculpting a behaviorally relevant representation", but just expressed differently?

The authors frequently refer to the FF for the Control subjects as "ambiguous" because they only sample it in one arm/hand configuration. However, in the way that they present the learning problem in the manuscript (e.g. ln 34-36, 173-174), one might actually expect that this ambiguity could put them at a disadvantage with respect to other subjects, especially the Cartesian group who experience it in 3 different hand configurations and can then better define its spatially-constant nature. Why then is the performance of the 2 groups essentially equivalent? Alternatively, maybe that result is not entirely surprising? It is not clear from the description of the task, but when the Cartesian subjects hold their hand in 3 different postures, do they also make reaching movements along slightly different compensatory spatial trajectories and correspondingly different shoulder/elbow joint rotations and torques, or do they in fact follow the same trajectories in shoulder/elbow joint velocity/torque space and the hand configurations have relatively little impact other than requiring small adjustments of forearm/wrist muscle activity to stabilize the wrist in the different postures? If it is the latter case, then the similar performance could be expected because the wrist postures are largely irrelevant to nature of the learning task. As a result, I don't understand why they state that the Cartesian field should be more difficult to learn if learning is in intrinsic coordinates because it is not evident how the change in hand posture impacts on the control of the shoulder and elbow joints (ln 398-401), which might actually be relatively constant across hand postures. This needs to be clarified.

The authors argue that the "Object" subjects learn the task because it is fixed in "object" space and so could be learnable. However, one could also consider this to be yet another demonstration that different hand or arm geometries provide sufficient "contextual" cues to permit simultaneous learning of multiple different and even anti-correlated FFs. The first such study that I can think of is Gandolfo et al 1996, but there have been several others in the ensuing years that the authors undoubtedly know better than me. This convergence of findings should be acknowledged in the manuscript. As for the slower learning rate, this may have to do with the fact that the FFs are in object-centered coordinates rather than Cartesian coordinates and so might be slightly more difficult to learn. Once again, however, if the reach trajectories are relatively constant in shoulder/elbow joint/torque space and most of the learning occurs in that coordinate framework, this means that the subjects only practice in each of the 3 FFs one third as often as the Control and Cartesian subjects, so slower learning should be expected. This point is not raised.

Finally, it is not too surprising that the Anti-object subjects showed the poorest learning because it is unlikely that any of themwould have ever encountered anything like these FFs that appear to contradict the usual laws of physics. On the other hand, the difference in learning between the Object and Anti-object groups demonstrates the limits of hand posture as a context cue to learn different FFs. This point should also be noted.

The trial conditions were varied in a pseudo-random fashion within task blocks. However, did they also vary randomly across subjects or did they present the same trial sequence to all subjects?

The first 2 pages of the Results section (ln 271-309) do not present a single result or data point. It is a summary of the task design that was already described in the Methods section. I acknowledge that it is very well written and clear, but it is not Results. Please incorporate this text into the Methods section.

Similarly, Fig 1A-D and Fig 2 are methodological in nature, while Fig 1E-L present results that fit more naturally with the data in Fig 3. Please restructure those figures more logically.
